# Case Report of Acute Esophageal Necrosis (Gurvits Syndrome) in Vaccinated, COVID-19-Infected Patient

**DOI:** 10.7759/cureus.22241

**Published:** 2022-02-15

**Authors:** Farrah Rahim, Sruthi Kapliyil Subramanian, Scott Larson

**Affiliations:** 1 Gastroenterology, University of Texas Health Science Center at Houston McGovern Medical School, Houston, USA

**Keywords:** black esophagus, esophagogastroduodenoscopy (egd), covid-19, gurvits syndrome, acute esophageal necrosis (aen)

## Abstract

Acute esophageal necrosis (AEN) is a rare endoscopic finding associated with ischemic compromise of the distal esophagus. This finding can be seen in critically ill patients with COVID-19 infection. We present a case of a COVID-19-vaccinated elderly male with multiple comorbidities and active COVID-19 pneumonia admitted to the intensive care unit with septic shock and acute hypoxemic respiratory failure. The patient developed melena, and esophagogastroduodenoscopy (EGD) was performed, which showed necrosis of the lower esophagus suggestive of AEN. AEN has been associated with high mortality and should be considered when evaluating upper gastrointestinal bleed in a critically ill patient. This case describes the first report of isolated AEN in a patient fully vaccinated against COVID-19 presenting with a severe complicated COVID-19 infection.

## Introduction

Acute esophageal necrosis (AEN), also known as “black esophagus” or Gurvits syndrome, is a rare condition with striking endoscopic findings characterized by diffuse, circumferential black discoloration of the distal esophagus [1,2]. It was first described by Goldenberg et al. in 1990 [3] and was identified as a distinct condition by Gurvits et al. in 2007 [1]. AEN occurs in critically ill patients with comorbidities and is associated with ischemia and low-flow states. It often presents with upper gastrointestinal (GI) bleeding [4]. From 1965 to 2006, only 88 cases of AEN were reported, and its pathophysiology continues to be poorly understood [1]. Complications of AEN include stricture formation and perforation, which can lead to mediastinitis, abscess formation, and potential sepsis [5]. Here, we describe a case of isolated AEN in a COVID-19-vaccinated patient presenting with a severe, complicated COVID-19 infection.

## Case presentation

A 74-year-old male with a history of hypertension, type II diabetes mellitus, and hyperlipidemia presented to the emergency department with diarrhea, generalized weakness, and dyspnea. He was admitted to the intensive care unit (ICU) for acute hypoxemic respiratory failure requiring mechanical ventilation and shock requiring vasoactive medications in the setting of severe complicated COVID-19 pneumonia, despite vaccination approximately six months prior. The type of vaccine is not known. At presentation, the patient had the following lab values table 1.

**Table 1:** Initial Laboratory Results K/uL = thousands per cubic milliliter; g/dL = grams per decilite; mmol/L = millimoles per lite; mg/dL = milligrams per decilite; ng/mL = nanograms per milliliter

Component	Lab Value	Reference Range
Blood urea nitrogen	88.0 mg/dL	7.0-25.0 mg/dL
Creatinine	3.4 mg/dL	0.7-1.3 mg/dL
White blood cell	17.3 K/uL	4.5-12.0 K/uL
Hemoglobin	13.3 g/dL	14.0-18.0 g/dL
Platelet	511 K/uL	150-400 K/uL
International normalized ratio	1.4	0.8-1.2
Sodium	138 mmol/L	136-145 mmol/L
Chloride	102 mmol/L	98-107 mmol/L
Bicarbonate	9 mmol/L	21-31 mmol/L
Anion gap	27 mmol/L	5-16 mmol/L
pH, venous	7.03	7.33-7.43
Lactic acid	10.5 mmol/L	0.5-2.0 mmol/L
C-reactive protein	38.9 mg/dL	< 10.0 mg/dL
Ferritin	6692.0 ng/mL	23.9-336.2 ng/mL

The hospital course was also complicated by acute kidney failure requiring hemodialysis and non-ST elevation myocardial infarction (NSTEMI) requiring initiation of aspirin, clopidogrel, and heparin (24.3 mL/hr).

**Figure 1:**
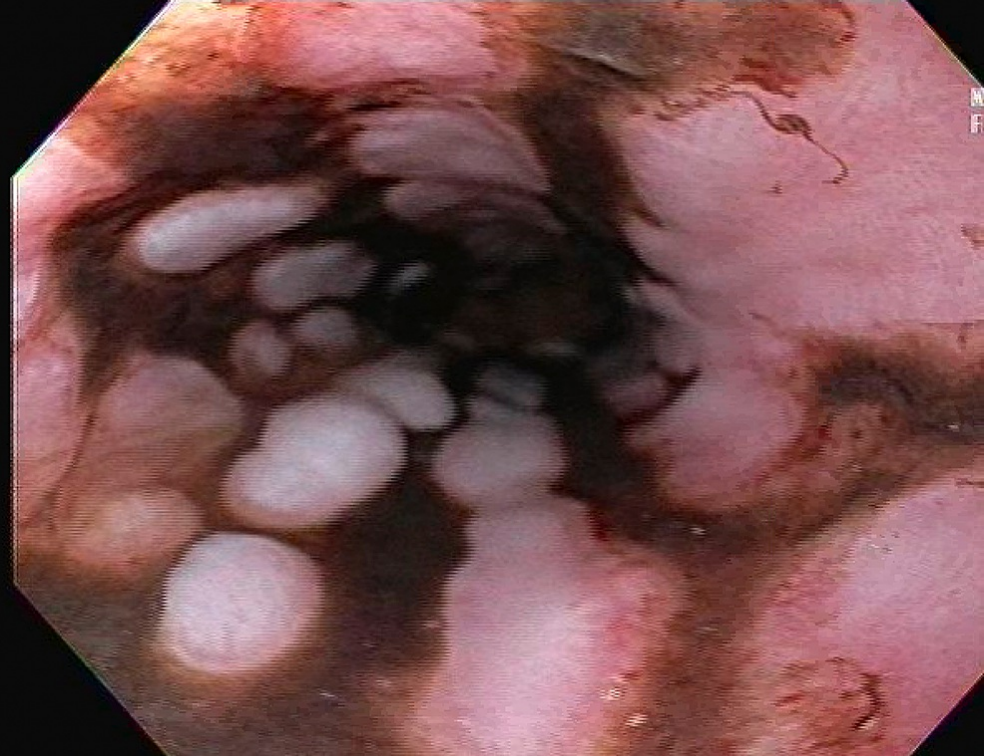
Patchy, circumferential, black discoloration of lower esophagus

**Figure 2:**
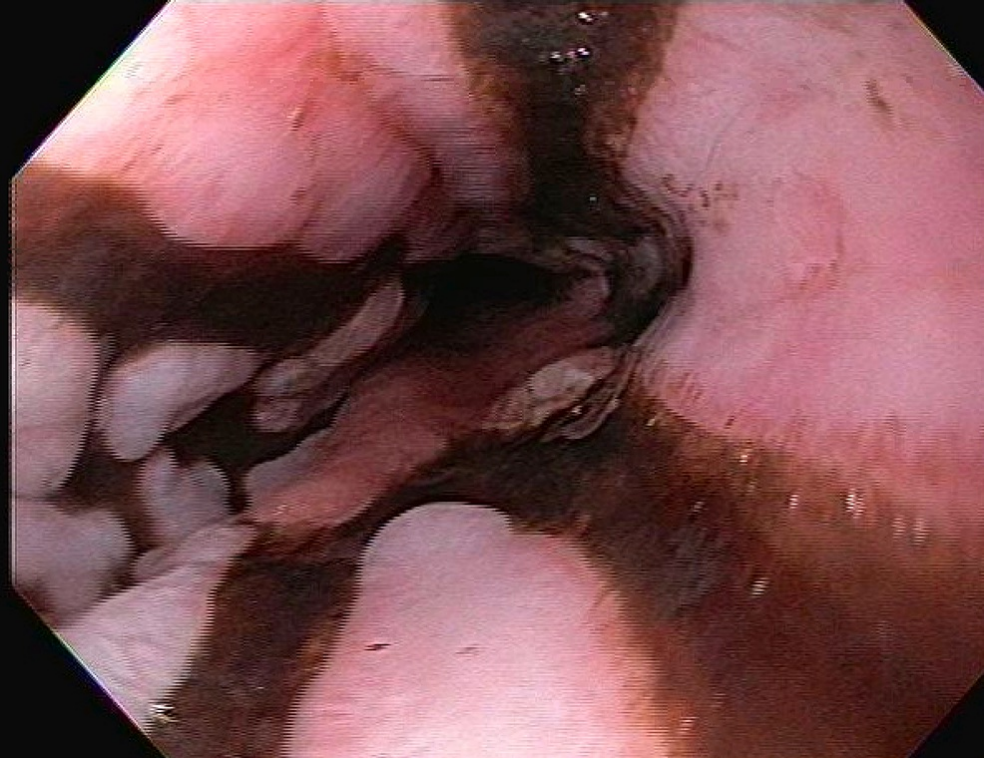
Patchy, circumferential, black discoloration of lower esophagus with distal red granulation tissue

On day five, the patient developed melena, and hemoglobin dropped to 8.6 g/dL from 13.3 g/dL at admission. Massive transfusion protocol and pantoprazole drip (8 mg/hr) were initiated. Esophagogastroduodenoscopy (EGD) was performed, which showed normal upper and middle esophagus. The lower esophagus showed extensive, patchy, circumferential, black discoloration with a sharp transition to normal esophageal tissue, suggestive of AEN (Figure 1, 2). Patchy, red granulation tissue (Figure 2) was also visualized, indicating resolving necrosis. No biopsies were obtained due to the extent of necrosis and characteristic appearance of AEN.

There was a small clean-based ulcer at the gastroesophageal junction with no active bleeding and multiple linear red spots on the lesser curvature, which appeared to be due to nasogastric tube suction trauma, with no active bleeding. The rest of the stomach was regular. The duodenal bulb showed duodenitis, and the rest of the duodenum was normal.

The patient had no further evidence of bleeding and was kept NPO and started on IV fluids and pantoprazole 40 mg IV twice daily. The patient was placed on vancomycin, meropenem, and micafungin for sepsis and was clinically managed for his COVID-19 infection. Unfortunately, on day eight of hospitalization, the patient died due to cardiac arrest in the setting of respiratory failure secondary to COVID-19 infection.

## Discussion

Acute esophageal necrosis is a rare condition often seen in critically ill patients. It is best diagnosed by EGD showing diffusely black esophagus with the abrupt transition of necrotic mucosa to normal tissue. AEN is caused by multiple factors, including decreased vascular flow, impaired mucosal barrier, and exposure to corrosive gastric content [4]. Ischemic or low-flow states can be associated with vasculopathy, hypotension, or shock.

AEN in COVID-19 infection has been described recently by Gurvits et al. A combination of COVID-related hypoperfusion and prothrombotic state and gastric reflux and chronic alcoholism contributed to this the development of AEN in the case described [6]. COVID-19 infection leading to thrombosis, as evidenced by the NSTEMI seen in our patient, in addition to the hypoperfusion state, can occur in vaccinated patients and appears to likely be the cause of AEN in our patient.

AEN is associated with nearly 32% mortality, though primarily attributed to underlying critical illness. The mortality specifically attributable to AEN is around six percent. AEN has a unique appearance on endoscopy, enough to establish the diagnosis, but some literature recommends a biopsy [7]. Others report that biopsy can increase the risk of perforation and is not necessary if the clinical and endoscopic findings are consistent with AEN [8]. Management is focused on treating the underlying illness and involves fluid resuscitation, gastric acid suppression, and parenteral nutrition when necessary. Antibiotic therapy in AEN is controversial, but appropriate antibiotic coverage should be provided to cover the specific pathogen identified in a biopsy specimen and other infectious causes associated with the development of the black esophagus [4].

## Conclusions

AEN is a rare condition with distinct endoscopic findings. Due to its rare findings and associated high mortality rates, identifying this condition and treating the underlying disease can help reduce mortality.
